# Oncostatin-M inhibits luteinizing hormone stimulated Leydig cell progenitor formation in vitro

**DOI:** 10.1186/1477-7827-5-43

**Published:** 2007-11-08

**Authors:** Katja J Teerds, Federica MF van Dissel-Emiliani, Maria P De Miguel, Mieke de Boer-Brouwer, Lina M Körting, Eddy Rijntjes

**Affiliations:** 1 Department of Biochemistry and Cell Biology, Faculty of Veterinary Medicine, Utrecht University, Utrecht, the Netherlands; 2 Department of Animal Sciences, Human and Animal Physiology Group, Wageningen University, Wageningen, the Netherlands; 3 Cell Engineering Laboratory, La Paz Hospital, Madrid, Spain

## Abstract

**Background:**

The initial steps of stem Leydig cell differentiation into steroid producing progenitor cells are thought to take place independent of luteinizing hormone (LH), under the influence of locally produced factors such as leukaemia inhibitory factor (LIF), platelet derived growth factor A and stem cell factor. For the formation of a normal sized Leydig cell population in the adult testis, the presence of LH appears to be essential.

Oncostatin M (OSM) is a multifunctional cytokine and member of the interleukin (IL)-6 family that also includes other cytokines such as LIF. In the rat OSM is highly expressed in the late fetal and neonatal testis, and may thus be a candidate factor involved in Leydig cell progenitor formation.

**Methods:**

Interstitial cells were isolated from 13-day-old rat testes and cultured for 1, 3 or 8 days in the presence of different doses of OSM (range: 0.01 to 10 ng/ml) alone or in combination with LH (1 ng/ml). The effects of OSM and LH on cell proliferation were determined by incubating the cultures with [3H]thymidine or bromodeoxyuridine (BrdU). Developing progenitor cells were identified histochemically by the presence of the marker enzyme 3beta-hydroxysteroid dehydrogenase (3beta-HSD).

**Results:**

OSM, when added at a dose of 10 ng/ml, caused a nearly 2-fold increase in the percentage of Leydig cell progenitors after 8 days of culture. Immunohistochemical double labelling experiments with 3beta-HSD and BrdU antibodies showed that this increase was the result of differentiation of stem Leydig cells/precursor cells and not caused by proliferation of progenitor cells themselves. The addition of LH to the cultures consistently resulted in an increase in progenitor formation throughout the culture period. Surprisingly, when OSM and LH were added together, the LH induced rise in progenitor cells was significantly inhibited after 3 and 8 days of culture.

**Conclusion:**

Taken together, the results of the present study suggest that locally produced OSM may not only play a role in the regulation of Sertoli cell proliferation and the initiation of spermatogenesis but may also play a role in the regulation of Leydig cell progenitor formation by keeping the augmenting effects of LH on this process in abeyance.

## Background

In the rat two defined periods of proliferation and differentiation of Leydig cells can be discerned. The first wave occurs during fetal life and gives rise to the fetal-type population of Leydig cells, while the second wave is initiated during the (pre)pubertal period and results in the formation of the mature adult-type Leydig cell population [1-3]. Between days 14 and 21 after birth the number of fetal-type Leydig cells starts to decrease, although 50 to 75% of the fetal-type Leydig cells present at the time of birth persist in the adult testis [4]. The second generation of Leydig cells, the so-called adult-type Leydig cells, develops from stem Leydig cells through several steps of differentiation and proliferation during (pre)puberty [2,3,5-8]. Spindle-shaped stem Leydig cells of mesenchymal origin, identified by the presence of platelet derived growth factor receptor α (PDGFR-α), leukemia inhibitory factor (LIF) receptor and c-kit, and the absence of LH receptors and steroidogenic enzyme expression, are thought to differentiate into luteinizing hormone (LH) receptor/3β-hydroxysteroid dehydrogenase (3β-HSD) positive Leydig cell progenitors between days 10 and 13 after birth. These progenitors undergo a wave of proliferation and differentiation and become immature adult-type Leydig cells between days 28 and 35 after birth. The immature Leydig cells subsequently differentiate into mature, terminally differentiated, adult-type Leydig cells. By the end of puberty the development of the adult population is completed. Each step in this developmental process is characterized by specific morphological aspects of the developing cells [3,7,9,10] and the expression of specific steroidogenic enzymes, such as 5α-reductase, 3β-HSD, cholesterol side chain cleavage (P450_scc_) and 17α-hydroxylase (P450_17a_) [11-13].

A considerable number of studies have been performed to investigate the regulation of this complicated developmental process in more detail [14-17]. Treatment of hypophysectomized prepubertal rats with highly purified LH has been shown to stimulate both differentiation of stem Leydig cells/precursor cells and proliferation of the newly formed progenitor Leydig cells [16]. Similarly, treatment of prepubertal boys with the luteinizing hormone (LH) analogue human chorionic gonadotropin (hCG) induces the formation of new Leydig cells through stem cell/precursor cell differentiation [15]. The importance of LH in this developmental process was recently further stressed in vitro [18]. In this study we showed, using interstitial cell preparations isolated from 10-, 13- or 18-day-old rat testes, that Leydig stem cells acquire first LH receptors and become precursor cells before they differentiate into LH receptor/3β-HSD positive progenitors. LH appears to be essential for this differentiation process to proceed, even at extremely low doses [18] to establish a normal sized mature Leydig cell population in the adult testis [19].

Although the above studies indicate that LH plays an important role in the formation of the adult-type Leydig cell population, there are indications that other factors may be involved in this process as well. Knockout studies have shown that in the absence of the Desert hedgehock (Dhh) gene [20] or platelet-derived growth factor-A (PDGF-A) [21], progenitor Leydig cell development did not occur in the prepubertal testis, despite plasma LH levels being normal. Whether these factors exert their effect on this developmental process alone or together with other factors, such as members of the interleukin family, is at present not known.

Oncostatin-M (OSM) is a polypeptide growth factor that was first identified in humans by its ability to inhibit solid tumour cell proliferation [22]. Amino acid analysis has demonstrated that OSM is a member of the interleukin-6 (IL-6) family of cytokines, which includes leukemia inhibitory factor (LIF), cilliary neurotrophic factor (CNTF) [23] and IL-11 [24]. All members of this family of cytokines act through high affinity heterodimer receptors that share the signal transducing receptor gp130 [25]. OSM mediates its bioactivity through two different heterodimer receptors, the type I OSM receptor which consists of a dimer of the gp130 transducing receptor and the LIF receptor β-subunit, and the type II OSM receptor in which gp130 has dimerized with the OSM receptor β-subunit [26]. In the rat OSM is highly expressed in the late fetal and neonatal testis, as well as in the maturing and adult testis though at a lower level [27]. In vitro studies have demonstrated that neonatal Sertoli cells produce OSM and that OSM stimulates Sertoli cell and gonocyte proliferation [28,29]. Preliminary observations showed that OSM immunoreactivity was also observed in the interstitial compartment of the developing testis, presumably in the Leydig cells in both rats [27] and humans [29].

In the present study the possible effects of OSM on the differentiation of spindle-shaped stem Leydig cells/precursor cells into 3β-HSD positive progenitor cells was investigated in more detail. Interstitial cells were isolated from 13-day-old rat testes, an age at which the first Leydig cell progenitors have just been formed in the sub-strain of Wistar rats used in this study, and cultured in the presence of LH and different doses of OSM. Differentiation of cells into Leydig cell progenitors was assessed by quantifying the presence of 3β-HSD positive cells under the different culture conditions. The proliferative activity of the cells was assessed by ^3^H-thymidine and BrdU incorporation.

## Methods

### Isolation of cells

Immature Wistar rats were obtained from the Central Animal Facilities of Utrecht University (the Netherlands). For each experiment groups of 20 to 30 animals were killed at the age of 13 days by CO_2_/O_2 _asphyxiation. For the in vivo detection of OSM type I receptor/LIF receptor three 13-day-old rats were used. Following fixation in Bouin's fluid for 24 h the testes were transferred to 50% ethanol for 24 h after which the testes were stored in 70% ethanol until embedding in paraffin. All experiments were carried out at least 3 times.

Interstitial cells were isolated as described by Teerds and colleagues [18]. In brief, testes were removed, decapsulated and subjected to enzymatic digestion in a shaking water bath, using 5 mg collagenase (type IV, 213 U/mg; Worthington Biochemical Corp, Freehold, NJ) in 20 ml Hanks buffered salt solution (HBSS; Gibco, Life Technologies, Grand Island, NY). Following enzymatic digestion HBSS was added until a total volume of 50 ml was reached and larger tubular fragments were allowed to settle under unit gravity. The supernatant was removed and smaller fragments and cell clumps were now allowed to settle. The supernatant was then collected, filtered and centrifuged. The pellet obtained was resuspended in 2 ml culture medium consisting of RPMI 1640 supplemented with penicillin (110 U/ml), streptomycin (100 μg/ml), 0.1% BSA and glutamine (2 mM; all substances were obtained from Gibco), followed by centrifugation. The supernatant was centrifuged again and the pellet was also resuspended in 2 ml culture medium. Both pellets were pooled. Cells were either seeded in 24-well plates on glass cover-slips at a density of 150,000 cells per well, or in 96-well plates at a density of 75,000 cells per well. After 3 h of culture, the medium was replaced in order to remove contaminating germ cells after which the cell isolates consisted of myoid cells, spindle-shaped mesenchymal-like cells, Sertoli cells, fetal-type Leydig cells and very low numbers of macrophages and Leydig cell progenitors. The spindle-shaped mesenchymal-like cells which in the testis can be found in a peritubular as well as a perivascular localization [3], are considered to be the stem cells/precursor cells of the Leydig cell progenitors [2,5,8]. The reason why we chose to use a relatively impure interstitial cell preparation was to include those cell types which have been shown in vivo to secrete paracrine factors known to influence the differentiation of stem Leydig cells, such as the Sertoli cell and peritubular/myoid cell derived growth factors PDGF A, transforming growth factor α (TGF-α)/epidermal-like growth factor (EGF), stem cell factor (SCF) and LIF [8].

Cells were cultured for either 1, 3, or 8 days at 37°C in air plus 5% CO_2 _in the presence of either 0 or 1 ng/ml ovine LH (NIH-LH-S20, Endocrinology Study Section of the National Institute of Health, Bethesda, MD), 0.01, 0.1, 1 or 10 ng/ml human recombinant OSM (Genzyme, Cambridge, MA), or in the presence of a combination of LH and different concentrations of OSM. These concentrations of OSM and LH were chosen based on the results of previous studies [18,27]. The culture medium was replaced every two days.

The described experiments were approved by the ethical committee for laboratory animal welfare of the Faculty of Veterinary Medicine, Utrecht University, Utrecht, the Netherlands.

### Scintillation counting

The effect of LH and OSM on the proliferation of the interstitial cell preparations was determined by incubating the cell cultures (96 well plates, 75,000 cells/well) with [^3^H]thymidine (2 μCi/ml; SA, 0.5 Ci/mmol; Amersham, Aylesbury, UK). Cells were isolated from 13-day-old rat testes and cultured for 1, 3 or 8 days in the presence of varying doses of OSM and/or 1 ng/ml LH. [^3^H]thymidine was added to the wells during the last 18 h of the culture period after which the cells were harvested onto glass fibre filters and the amount of incorporated radioactivity was determined using a liquid scintillation analyser (Beta plate, LKB, Turku, Finland).

### 3β-HSD enzyme histochemistry and cell counts

Interstitial cells isolated from 13-day-old rat testes were cultured on glass cover-slips in the presence of LH and varying concentrations of OSM in 24 well plates. After 1, 3 or 8 days of culture, 3β-HSD activity was determined in the cultures according to the method of Loyda *et al*. [30] with minor modifications [31]. On each cover-slip a total of at least 1000 cells was counted (3β-HSD positive plus negative cells), using a Nikon Optiphot 2 microscope, equipped with differential interference contrast optics. The number of blue stained 3β-HSD positive cells was expressed as percentage of the total number of cells counted per cover-slip. In control experiments in which the addition of the substrate 5α-androstane-3β-ol-17-one was omitted, no blue staining was observed.

### Immunohistochemistry

#### Localization of OSM and OSM type I receptor/LIF receptor

For the localization of OSM interstitial cells were isolated, plated on glass cover-slips in 24 well plates and cultured for 3 or 8 days without any additions or in the presence of LH (1 ng/ml) and/or OSM (10 ng/ml). In case of the localization of OSM and OSM type I receptor/LIF receptor 5 μm thick sections were cut from paraffin embedded testes of 13-day-old rats. Detection was carried out as described by De Miguel et al. [27] with minor modifications. Briefly, cells were fixed in buffered formalin (4% formalin in 0.1. M PBS), rinsed in 0.01 M PBS, blocked with 10% normal goat serum and incubated with a polyclonal antibody against OSM or against OSM type I receptor/LIF receptor (Santa Cruz Biotechnology, Tebu-Bio, Heerhugowaard, the Netherlands), both diluted 1:100 in PBS to which 0.05% acetylated BSA (BSA-c, Aurion, Wageningen, the Netherlands) was added, for 2 h at room temperature (OSM) or overnight at 4°C). Cells were rinsed with PBS and incubated with a secondary goat-anti-rabbit biotinylated antibody (Vectastain Kit Elite, Vector Laboratories, Burlingame, CA, USA) diluted 1:100 in PBS/BSA-c for 1 h at room temperature, rinsed and incubated with the components A (avidin) and B (biotin) (Vector Laboratories) diluted 1:1000 in PBS/BSA-c. Labeling was visualized using DAB (Sigma). In control experiments the primary OSM antibody was replaced by normal rabbit serum. Cells were counter-stained using Mayer's hematoxylin and mounted with Aquatex (Merck, Darmstadt, Germany).

#### BrdU and 3β-HSD double labeling

In order to determine whether the 3β-HSD positive cells were also proliferating, interstitial cells were isolated, plated on glass cover-slips and cultured for 1 or 3 days without any additions or in the presence of OSM (10 ng/ml) and/or LH (1 ng/ml). Three hours before termination of the experiment BrdU (3.3 μg/ml, Sigma) was added to the cultures. Cells were fixed in methacarn (60% methanol, 30% chloroform, 10% acetic acid) for 10 min and then stored at 4°C in 70% alcohol until further processing. BrdU is incorporated in DNA of cells in the S-phase of the cell cycle and thus is a marker for the proliferative activity of cells. Immunohistochemical labeling was carried out as described before [32] with minor modifications. Briefly, cells were pretreated with periodic acid prior to incubation with the monoclonal antibody against BrdU (Becton Dickinson Immunocytometry Systems, San Jose, CA) diluted 1:100 in PBS for 2 h at room temperature, rinsed and incubated with a peroxidase-conjugated rabbit-anti-mouse antibody (Sigma) diluted 1:100 in PBS. Labeling was visualized using DAB (Sigma) enhanced by nickel that gives a black precipitate. After this cells were extensively rinsed with PBS and incubated overnight at 4°C with a polyclonal antibody against 3β-HSD (gift from dr. Mason, Edinburgh, Scotland) as described before [33]. In brief, cells were incubated with the 3β-HSD antibody (diluted 1:300 in PBS/BSA-c) at room temperature, rinsed in PBS and incubated with a biotinylated goat-anti-rabbit antibody (Vector Laboratories) diluted 1:200 in PBS/BSA-c at room temperature. Next cells were rinsed and incubated with the components A (avidin) and B (biotin) (Vector Laboratories) diluted 1:1000 in PBS/BSA-c. Labeling was visualized using DAB (Sigma).

#### Statistical analysis

Data from cultures that had undergone the same treatment in different experiments were combined. Statistical analysis was performed to test whether the error variance of the dependent variable was equal across groups using Levene's test of equality of error variances. Statistical significance was assessed by using ANOVA and Bonferroni in case of multiple comparisons. Differences were considered to be significant when P < 0.05. The experiments were performed in triplicate or quadruplicate and repeated at least three times using different cell preparations. Data are expressed as mean ± SD.

## Results

### ^3^H-Thymidine incorporation

**Figure 1:**
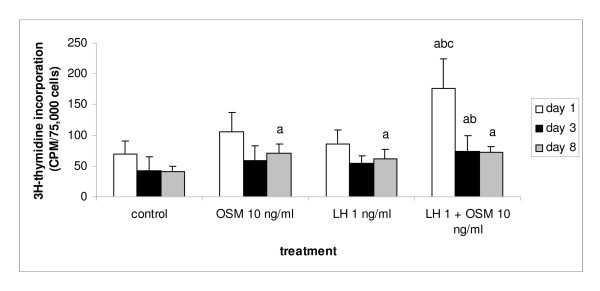
Effects of OSM (10 ng/ml) either alone or in combination with LH (1 ng/ml) on the proliferation of interstitial cells isolated from 13-day-old rat testes and cultured for 1, 3 or 8 days. Proliferation was determined by [^3^H]-thymidine incorporation and scintilation counting (see Materials and Methods). Statistical analysis revealed that there was no significant difference among the different experiments. Therefore, the results are represented as mean ± SD of three different experiments that were combined. Each single experiment was carried out in quadruplicate. Differences among groups were considered to be significantly different when p < 0.05. a – significantly different from control; b – significantly different from OSM 10 ng/ml; c – significantly different from LH 1 ng/ml.

OSM, a cytokine locally produced in the testis, has been shown to stimulate gonocyte proliferation in the neonatal testis of rats mice and humans [27,34]. In the present study it was tested whether OSM also has a growth stimulating effect on interstitial cells isolated from 13-day-old rat testes. Cells were cultured for different periods of time in the presence of increasing concentrations of OSM (ranging from 0.01 to 10 ng/ml). Independent of the length of the culture period, the three lowest concentrations of OSM (0.01, 0.1 and 1 ng/ml) did not significantly influence [^3^H]-thymidine incorporation by the interstitial cells, compared to the control cultures (data not shown). OSM at a concentration of 10 ng/ml had only a stimulating effect on [^3^H]-thymidine incorporation after 8 days of culture compared to the control cells that were cultured for the same length of time (Figure 1). Addition of LH to an OSM dose of 10 ng/ml resulted in an increase of [^3^H]-thymidine incorporation after 1 day of culture. After 3 days of culture the level of [^3^H]-thymidine incorporation was still higher than in the cultures treated with OSM alone. By 8 days of culture this effect was no longer seen, though the level of [^3^H]-thymidine incorporation remained higher when compared to the control cells cultured for 8 days (Figure 1).

### 3β-HSD enzyme histochemistry and cell counts

The next step was to investigate whether OSM also affected the differentiation of Leydig stem cells/precursor cells into progenitor cells. For this experiment the dose of 10 ng/ml OSM was chosen. The percentage of 3β-HSD positive cells was counted as an indicator of the development of Leydig cell progenitors.

**Figure 2:**
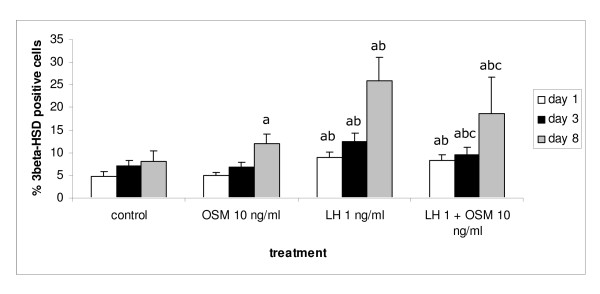
Effect of OSM (10 ng/ml) either alone or in combination with LH (1 ng/ml) on the presence of 3β-HSD positive Leydig cell progenitors. Interstitial cells were isolated from 13-day-old rat testes and cultured for 1, 3 or 8 days. The presence of 3β-HSD was determined by enzyme histochemistry or immunohistochemistry using a polyclonal antibody against 3β-HSD (for details see Materials and Methods). Statistical analysis revealed that there was no significant difference among the different experiments and thus the results are presented as mean ± SD of three different experiments that were combined. Each single experiment was carried out either in duplicate, triplicate or quadruplicate. Differences among groups were considered to be significantly different when p < 0.05. a – significantly different from control; b – significantly different from OSM 10 ng/ml; c – significantly different from LH 1 ng/ml.

In freshly isolated interstitial cell preparations from testes of 13-day-old rats, the percentage of 3β-HSD positive cells was less than 2% (data not shown). At this stage the 3β-HSD positive cell population consisted of a mix of fetal-type Leydig cells and some newly developed Leydig cell progenitors. After 1–3 days of culture without any additions the percentage of 3β-HSD positive cells had increased about 2-to 4-fold, confirming that these cells underwent spontaneous differentiation (Figure 2) [18]. Addition of 10 ng/ml OSM to the cells resulted after 8 days of culture in a slight but significant increase in the percentage of 3β-HSD positive progenitor cells compared to the control cells cultured for the same length of time (Figure 2). LH administration resulted throughout the culture period in higher percentages of 3β-HSD positive progenitors compared to the controls and the cells cultured in the presence of OSM for the same period of time (Figure 2). Surprisingly, when OSM and LH were administered together the percentage of 3β-HSD positive cells was after 3 and 8 days of culture significantly lower compared to the cells cultured in the presence of LH alone, though still significantly higher compared to the cells cultured in the presence of OSM alone and the respective control cultures (Figure 2).

#### BrdU and 3β-HSD double labeling

The next question that arose was, whether the observed increases in 3β-HSD positive cells following OSM were the result of differentiation of stem Leydig cells/precursor cells into 3β-HSD positive progenitors, or proliferation of the 3β-HSD positive cells already present in the cultures. In order to investigate this, interstitial cells were isolated and cultured for 1 or 3 days in the presence of OSM (10 ng/ml) or LH alone, or in the presence of the combination of OSM and LH. Before terminating the experiments, cells were incubated with BrdU (for details see Materials and Methods) to determine the proliferative activity of the cells. Cells were double labeled with antibodies against BrdU and 3β-HSD. The results of this experiment showed that the percentage of double labeled cells following OSM treatment alone or in combination with LH was neither after 1 day nor after 3 days of culture significantly different from zero, and also did not differ significantly from the controls (day 1: 0.1 ± 0.1 (OSM), 0.2 ± 0.2 (LH + OSM), 0.1 ± 0.1 (control); day 3: 0 (OSM), 0.9 ± 1.3 (LH + OSM), 0.1 ± 0.1 (control). After 8 days of culture no BrdU/3β-HSD double stained positive cells could be detected under any of the culture conditions. As has been reported previously, LH alone did not influence the proliferative activity of the 3β-HSD positive cells under the present culture conditions (18).

### OSM and OSM type I receptor/LIF receptor immunohistochemistry

**Figure 3:**
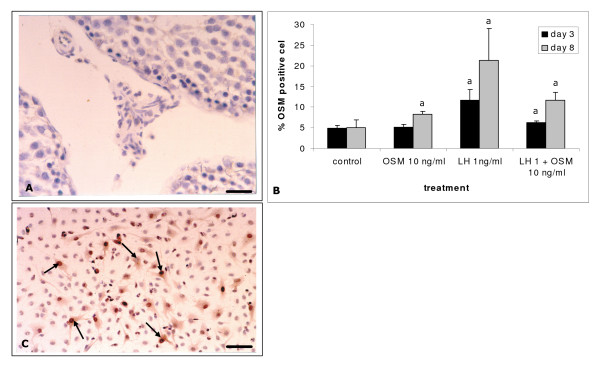
Immunohistochemical labelling for the presence of OSM in vivo and in vitro. A) Section of the testis of a rat 8 days after EDS administration. All Leydig cells have disappeared from the interstitium. Under these conditions no OSM positive cells could be detected by immunohistochemistry; B) Interstitial cell were isolated from testes of 13-day-old rats and cultured for 3 or 8 days without any additions or in the presence of 10 ng/ml OSM and/or 1 ng/ml LH (for more details see Materials and Methods). Percentage of OSM positive cells in culture determined under the indicated culture conditions. The experiment was carried out in quadruplicate. Values are represented as mean ± SD; statistical analysis was carried by comparing the treated cultures with their respective controls. Means were considered to be significantly different at p < 0.05 (indicated by (a)). C) Cells cultured in the presence of LH for 8 days and immunohistochemically stained with an antibody against OSM. Some positively stained cells are indicated by arrows. Scale bar in (A) represents 21 μm, in (C) 20 μm.

De Miguel and colleagues [27,29] have reported that in the developing and adult testis interstitial cells, presumably Leydig cells, stained positively with an antibody against OSM. In the present study the nature of the OSM positive cells was confirmed by immunohistochemical staining of testis sections derived from adult rats treated with the Leydig cell toxicant ethane dimethyl sulphonate (EDS). This drug specifically destroys Leydig cells in the adult testis, a process followed by the formation of a complete new population of Leydig cells [e.g. [35]]. This regeneration process has many similarities with the development of the adult-type Leydig cell population in the prepubertal rat [36]. Eight days after EDS administration at a point in time when Leydig cells had not yet started to repopulate the testis, no OSM positive interstitial cells could be detected in the interstitial compartment (Figure 3A). This suggests that the OSM positive interstitial cells in the study by De Miguel et al. [27] were indeed Leydig cells.

Analysis of cells cultured for 3 or 8 days in the presence of LH and stained immunohistochemically for the presence of OSM, showed a significant increase in the percentage of OSM positive cells when compared to the untreated controls cultured for the same periods of time (Figure 3B, C). When OSM was given together with LH the stimulating effect of LH was partially inhibited (Figure 3B).

**Figure 4:**
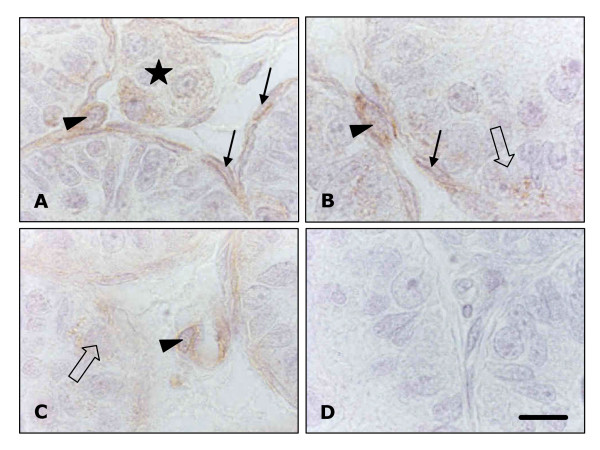
Immunohistochemical labelling for the presence of LIF-R in the testis of 13-day-old rats (A-C). Faint brown staining for the presence of OSM type I receptor/LIF receptor is present in a cluster of fetal-type Leydig cells (A; asterisk). The staining is much stronger in stem Leydig cells/precursor cells identified by their spindle-shaped nucleus, located in close vicinity of the basal membrane of the seminiferous tubules (A, B; filled arrows) and in Leydig cell progenitors identified by their oval nucleus which are either found close to the seminiferous tubules (A, B) or blood vessels (C; arrowhead). Staining in spermatogonia was faint (B, C; open arrow). (D) Control in which the first antibody was replaced by rabbit serum. Scale bar represents 11 μm.

Immunohistochemical staining for the presence of OSM type I receptor/LIF receptor of paraffin embedded sections of testes from 13-day-old rats revealed that in the interstitium this receptor was present on spindle-shaped peritubularly located stem Leydig cells/precursor cells, as well as on progenitor cells and fetal-type Leydig cells (Figure 4). The number of positively stained macrophages was very small due to the low numbers of macrophages present in the 13-day-old rat testis (not shown). Within the seminiferous tubules the spermatogonia stained faintly positive (Figure 4). No staining was observed in the Sertoli cells, presumably because the amount of antigen present was below the detection limit of the immunohistochemical assay.

## Discussion

Although Leydig cells have been shown to possess LIF receptors [37] it is not known whether these cells or their stem cells/precursor cells express functional OSM receptors and are thus able to respond to OSM released by for instance Sertoli cells. The present study is the first to demonstrate that these stem cells/progenitor cells express the LIF receptor/OSM type 1 receptor and that OSM by binding to this receptor can affect the formation of Leydig cell progenitors in vitro. Nevertheless, the effects of OSM on progenitor development seem to be contradictory, depending on the presence of LH. When interstitial cells were cultured in the presence of OSM for a prolonged period of time, progenitor formation was increased by approximately 40%, due to differentiation of stem Leydig cells/precursor cells. However, when the cells were cultured in the presence of the combination of OSM and LH, OSM appeared to significantly inhibit the LH stimulated formation of progenitor cells by about 35%.

We have reported recently that when testicular interstitial cells were isolated from 13-day-old rats and cultured, a small subpopulation of stem Leydig cells/precursor cells can develop into Leydig cell progenitors autonomously within 3 days after plating, independent of the immediate presence of LH [18]. Since OSM is highly expressed in the fetal and neonatal rat testis, we hypothesized that OSM may be a candidate factor involved in the autonomous formation of progenitor cells. This hypothesis is, however, not supported by the results of the present study, since it takes between 4 and 8 days for OSM to augment the number of progenitor cells, making it unlikely that OSM plays a role in the autonomous formation of progenitor cells.

Incubation of interstitial cells with the combination of LH and OSM, did not lead to a synergistic effect on progenitor formation. On the contrary, the augmenting effect of LH on the differentiation of stem Leydig cells/precursor cells into 3β-HSD progenitor cells after 3 and 8 days of culture was partially inhibited by OSM. LH acts though activation of a G-protein coupled receptor inducing the formation of the second messenger cAMP, in general followed by activation of PKA, tyrosine phosphorylation and activation of Ras [38]. OSM binding to its receptor complex, the type I or type II OSM receptor, activates the Janus tyrosine kinases (Jaks) and the activated Jaks in turn activate downstream pathways such as SHP-2 tyrosine phosphatase and STAT3 [39]. How these two signal transduction pathways communicate with each other, resulting in the partial inhibition of LH stimulated progenitor formation is at present unknown.

Several studies have reported on a possible physiological role of OSM in testicular development. In co-cultures of rat Sertoli cells and gonocytes human recombinant OSM appeared to stimulate gonocyte proliferation [27], while in mouse derived neonatal testicular cell cultures, recombinant mouse OSM was shown to enhance Sertoli cell proliferation [34]. Furthermore, Sertoli cells have been shown to release an OSM-like protein, implicating that under in vivo conditions locally produced OSM may affect both neonatal Sertoli cell proliferation and be involved in the initiation of spermatogenesis [27]. Over-expression of OSM in transgenic mice, on the other hand, leads to developmental abnormalities and infertility due to the absence of developing spermatocytes [40]. These authors further hypothesize that in order for OSM to exert a regulatory role on early testicular development, its synthesis and release need to be tightly regulated. The present study extends these observations by showing that OSM does not only affect Sertoli cells and germ cells in the neonatal testis, but possibly also plays a role in the formation of the adult Leydig cell population by keeping the stimulating effect of LH on Leydig cell progenitor formation in abeyance.

The combination of LH plus OSM not only affected progenitor formation but also induced an enhancement of [^3^H]-thymidine incorporation compared to the control group, especially after 1 day of culture. Since the percentage of 3β-HSD/BrdU double labeled cells had not increased under these conditions, it is postulated that these proliferating cells are not Leydig cell progenitors. However, since Leydig cells and their precursors are the only cell types in the interstitium that express functional LH receptors [18], the effects on cell proliferation induced by the combination of LH and OSM, are most likely mediated indirectly. Possibly, precursor cells and/or progenitor cells are stimulated to release (growth) factors that affect the proliferative activity of other cells present in the cultures.

Several studies have reported the presence of OSM positive cells in the testicular interstitium in different species such as rat, mouse, monkey and human. These authors suggested that the OSM expressing cells in the interstitium may be Leydig cells [27,29,41]. In the present study immunohistochemical analysis revealed that the effects of OSM and/or LH on the percentage of OSM positive cells showed a consistent similarity to the effects of these factors on the percentage of 3β-HSD positive progenitor cells, implicating that the OSM positive cells may indeed be Leydig cells. This was confirmed by treatment of rats with the Leydig cell toxicant EDS, which specifically destroys Leydig cells in the adult testis [36]. Following EDS administration, no cells positively stained for the presence of OSM could be detected in the interstitium by immunohistochemistry.

Many of the described effects of OSM overlap with the effects of LIF a highly pleiotropic cytokine. As indicated above, OSM can bind to the LIF receptor β subunit dimerized with gp130, the so-called type I OSM receptor as well as to the type II OSM receptor, consisting of a dimer of gp130 and the OSM-specific receptor β subunit (OSMR) [25,26]. Sertoli cells but also Leydig cells and spermatogonia, have been reported to express functional LIF receptors [28,42], which may explain why targeted disruption of the type II OSM receptor [40], did not lead to infertility. On the other hand, in a recent study Kanatsu-Shinohara and colleagues (2007) showed that while LIF enhanced the formation of germ cell colonies in neonatal mouse testis cultures, OSM failed to exert this effect, suggesting that OSM can not replace LIF in all cases and that at least in part these molecules have differential effects on spermatogonial development [43].

## Conclusion

Taken together, the results of the present study suggest that locally produced OSM is not only involved in the regulation of Sertoli cell proliferation and the start of spermatogenesis, but may also play a role in the regulation of Leydig cell progenitor formation by keeping the stimulating effects of LH on this process in abeyance.

## Competing interests

The author(s) declare that they have no competing interests.

## Authors' contributions

KJT participated together with FMFvD-E in the design of the study. The experiments were carried out by MdB-B, MPdM and LMK. Data analysis was performed by KJT, FMFvD-E, MPdM and ER. ER helped to draft the manuscript; the manuscript was written by KJT. All authors have read and approved the final manuscript.
